# First report and new molecular and morphological characterizations of root-knot nematode, *Meloidogyne javanica*, infecting ginger and long coriander in Vietnam

**DOI:** 10.21307/jofnem-2021-011

**Published:** 2021-02-15

**Authors:** Ke Long Phan, Thi Mai Linh LE, Huu Tien Nguyen, Thi Duyen Nguyen, Quang Phap Trinh

**Affiliations:** 1 Vietnam National Museum of Nature, Vietnam Academy of Sciences and Technology, 18 Hoang Quoc Viet, Cau Giay, 100000, Hanoi, Vietnam; 2 Institute of Ecology and Biological Resources, Vietnam Academy of Sciences and Technology, 18 Hoang Quoc Viet, Cau Giay, 100000, Hanoi, Vietnam; 3 Graduate University of Science and Technology, Vietnam Academy of Sciences and Technology, 18 Hoang Quoc Viet, Cau Giay, 100000, Hanoi, Vietnam; 4 Nematology Research Unit, Department of Biology, Ghent University, K.L. Ledeganckstraat 35, 9000, Ghent, Belgium

**Keywords:** 28S rRNA, ITS, mtDNA, DNA barcode, Multiplex-PCR, Taxonomy, Plant-parasitic nematodes

## Abstract

Ginger (*Zingiber officinale* Roscoe) and long coriander (*Eryngium foetidum* L.) are commonly grown and used as important spices and medicinal plants in Vietnam. Our study recovered for the first time one of the most damaging tropical root-knot nematodes, *Meloidogyne javanica*, associated with these plants in the Western Highlands of Vietnam. In this study, *M. javanica* was characterized based on morphology and molecular characterization of D2-D3 fragment of 28S rRNA, ITS, and *Nad5* mtDNA regions. The identification of this species was done based on a combination of morphology, multiplex-PCR with specific primer, network haplotype analysis, and PPNID program.

Root-knot nematodes belonging to the genus *Meloidogyne* are one of the most damaging plant-parasitic nematodes of the world (Jones et al., 2013). These nematodes parasitize thousands of plant species and cause significant yield loss (Agrios, 2005; CABI, 2020; Jones et al., 2013). Among more than 100 known species, the tropical (*Meloidogyne arenaria* (Chitwood, 1949; Neal, 1889), *Meloidogyne incognita* (Chitwood, 1949; Kofoid and White, 1919), and *Meloidogyne javanica* (Chitwood, 1949; Treub, 1885) and the temperate (*Meloidogyne hapla*, Chitwood, 1949) root-knot nematodes are the most economically important and commonly known species of the world (Jones et al., 2013; Kazachenko and Mukhina, 2013; Nguyen et al., 2019). On ginger, *Zingiber officinale* Roscoe, six species of the genus *Meloidogyne* have been reported including *M. arenaria*, *M. hapla*, *M. incognita*, *M. javanica*, *M. thailandica*, and *M. enterrolobii* (Handoo et al., 2005; Sikora et al., 2018; Xiao et al., 2018), of which, *M. incognita* seems to be the most frequently reported nematode associated with ginger (Myers et al., 2017). Especially, *Meloidogyne javanica* has been found associated with ginger in several countries, including the United States, India, and Brazil (Baptista Dos Santos and Lazaro Lozano, 1993; Hajihassani et al., 2019; Singh and Gupta, 2011). However, only *M. incognita* has been reported on long coriander (*Eryngium foetidum* L.) in India (Sheela et al., 2003). In Vietnam, *M. javanica* has been identified based on morphological features only (Nguyen and Nguyen, 2000), except for the recent report of *M. javanica* on black pepper (*Piper nigrum* L.) applied specific primers in identifying this species (Nguyen et al., 2020).

In the past, *Meloidogyne* species were identified using morphological and morphometric characterizations, and consequently, a large number of *Meloidogyne* spp. were described without molecular data (Karssen, 2002; Perry et al., 2009). Although molecular approach has facilitated the identification process of root-knot nematodes, both morphological and molecular characterizations showed that the three most important tropical root-knot nematodes are very closely related (Álvarez-Ortega et al., 2019; Perry et al., 2009). Remarkably, the study of Janssen et al. (2016) proved that some mtDNA genes, especially *Nad5* mtDNA gene, are strongly linked with traditional esterase isozyme patterns, and therefore, it can be used as an efficient barcode marker for the reliable identification of tropical root-knot nematodes.

In this study, morphology and morphometric of second-stage juveniles, males, and females of *Meloidogyne javanica* on ginger and long coriander in Vietnam were provided for the first time. The study revealed new distribution and new host for *M. javanica.* Besides, molecular characterization of D2-D3 fragment of rDNA, ITS rDNA, and *Nad5* mtDNA gene regions were given to support the morphological identification of *M. javanica* populations in this study.

## Material and methods

Soil and root samples were collected from the upper 30 cm soil layer in the growing areas of ginger (*Zingiber officinale* Roscoe) and long coriander (*Eryngium foetidum* L.) from the Western Highlands in Vietnam. Vermiform nematodes were extracted using the modified Baermann tray method (Whitehead and Hemming, 1965) and swollen mature females were extracted directly from the galls with a stereomicroscope, using a scalpel and forceps (Perry et al., 2009). Subsequently, nematodes were fixed and prepared to make permanent slides following Nguyen et al. (2019). For morphological characterization, measurements and pictures were taken from permanent slides using Carl Zeiss Axio Lab. A1 light microscope equipped with a Zeiss Axiocam ERc5s digital camera. For molecular characterization, Multiplex-PCR using primers Mi2F4/Mi2R1, Far/Rar, and Fjav/Rjav was performed following Kiewnick et al. (2013) to quickly identify *M. javanica* from closely related species in the tropical root-knot nematode group. The D2-D3 region of 28S rRNA, ITS, and *Nad5* mtDNA gene regions were amplified using D2A/D3B, Vrain2F/Vrain2R, and NAD5F2/NAD5R1 primers (Nguyen et al., 2019; Trinh et al., 2019). Forward and reverse sequences were assembled using Geneious R11 (www.geneious.com). The BLAST search was used to check for the similarities with other related sequences on GenBank (Altschul et al., 1997). Alignment between our *Nad5* mtDNA sequences and 73 reference sequences in the study of Janssen et al. (2016) was created using Muscle in Geneious R11. Median-joining network in POPART 1.7 was used to create a haplotype network from the alignment (Bandelt et al., 1999; Leigh and Bryant, 2015). PPNID program was used to confirm the identification of these populations based on *Nad5* mtDNA sequences (Qing et al., 2020).

## Results

### Measurements

(Table 1)

**Table 1.** Morphometric data of *Meloidogyne javanica* from different populations.

	Population from ginger in Vietnam		Population from long coriander in Vietnam		Population from sugar cane in Indonesia (Whitehead, 1968)		Population from tomato in Iran (Ghaderi et al., 2020)	
Diagnostic characters	J2	Female	J2	Female	J2	Female	J2	Female
*n*	20	10	20	10	25	20	50	15
*L*	395 ± 18.7 (341-418)	693 ± 110 (570-882)	402 ± 42 (300-521)	554 ± 31 (522-584)	417 ± 22 (387-459)	657 ± 67 (541-804)	464 ± 19 (429-497)	686 ± 127 (506-1.01)
Lip height	2.6 ± 0.2 (2.2-2.9)	2.7 ± 0.6 (1.9-3.9)	2.9 ± 0.4 (2.2-4.1)	3.3 ± 0.3 (2.9-3.6)	–	–	–	–
Lip width	3.7 ± 0.4 (3.2-4.7)	6.3 ± 1.2 (4.6-8.6)	4.1 ± 0.4 (3.2-5.0)	6.2 ± 0.9 (5.2-7.7)	–	–	–	–
Stylet	11.3 ± 0.8 (9.9-12.5)	16.2 ± 0.7 (15.1-17.2)	11.0 ± 0.8 (9.0-13.2)	15.0 ± 1.7 (12.6-17.3)	10.4 ± 0.5 (9.4-11.4)	15 (14-18)	10 ± 0.5 (9-12)	15 ± 2 (13-18)
DGO	3.0 ± 0.4 (2.4-3.8)	5.1 ± 1.2 (3.0-6.6)	3.3 ± 0.6 (2.4-5.3)	6.1 ± 0.5 (5.5-6.6)	–	3 (2-5)	2.2 ± 0.5 (1.5-3.7)	3.1 ± 0.6 (2.0-3.9)
Distance from anterior end to median bulb	54.3 ± 2.5 (50-59)	–	53 ± 6.8 (39-74)	–	–	–	–	–
Distance from anterior end to pharyngo-intestinal junction	84 ± 5.0 (76-92)	–	76 ± 10.7 (53-98)	–	–	–	–	–
Distance from anterior end to excretory pore	82 ± 6.5 (65-94)	34 ± 3.2 (29-40)	80 ± 8.0 (60-98)	38 ± 5.8 (34-48)	–	–	–	190 ± 30 (145-270)
Maximum body diameter	14.3 ± 0.8 (12.7-15.9)	445 ± 1,123 (269-602)	13.1 ± 1.8 (8.8-17.2)	271 ± 35 (237-306)	–	431 ± 63 (311-581)	15 ± 1.5 (12-18)	420 ± 99 (274-568)
Body diameter at anus	10.3 ± 0.8 (9.0-11.2)	–	9.7 ± 1.0 (7.3-12.0)	–	–	–	–	–
Tail length	50 ± 4.2 (42-57)	–	48 ± 6 (31-63)	–	49 ± 4 (36-56)	–	55 ± 5 (40-66)	–
Hyaline	13 ± 2.2 (9.1-17)	–	13.7 ± 2.0 (9.1-17)	–	–	–	13 ± 4 (7-20)	–
Distance from anterior end to end of pharynx	165 ± 14.6 (135-183)	–	150 ± 20 (103-210)	–	–	–	193 ± 23 (90-216)	–
*a*	28 ± 1.8 (24-32)	1.6 ± 0.3 (1.2-2.1)	31.1 ± 4.9 (22-50)	2.1 ± 0.1 (1.9-2.2)	30.6 ± 2.07 (27.1-35.9)	–	31 ± 3 (25-39)	1.7 ± 0.4 (1.2-2.6)
*b*	4.8 ± 0.4 (3.7-5.4)	–	5.4 ± 0.6 (3.7-7.2)	–	2.42 ± 0.291 (2.1-3.35)	–	5.2 ± 1.4 (4.3-12.4)	–
*b′*	2.4 ± 0.3 (2.0-3.1)	–	2.7 ± 0.4 (2.0-3.6)	–	–	–	2.5 ± 0.5 (2.0-5.2)	–
*c*	8.0 ± 0.6 (7.3-9.4)	–	8.4 ± 1.0 (6.8-10.8)	–	8.5 ± 0.68 (7.3-11.1)	–	8.5 ± 0.8 (7.2-11.3)	–
*c′*	4.8 ± 0.5 (3.8-5.9)	–	4.9 ± 0.6 (3.8-6.9)	–	–	–	5.7 ± 0.5 (4.7-7.2)	–
Neck length	–	204 ± 50 (139-279)	–	272 ± 36 (235-307)	–	–	–	259 ± 87 (153-540)
Ratio of body length to length of neck	–	3.6 ± 0.8 (2.0-4.6)	–	2.1 ± 0.2 (1.9-2.2)	–	–	–	–
Vulva slit length	–	18.4 ± 1.9 (15.6-21.6)	–	19.8 ± 1.0 (18.2-20.9)	–	–	–	21 ± 3 (15-27)
Vulva–anus distance	–	15.2 ± 1.0 (13.1-16.2)	–	16.4 ± 1.0 (15.6-18.0)	–	–	–	16 ± 4 (12-28)
Anus-tail tip distance	–	12.3 ± 2 (9.3-15.8)	–	10.6 ± 2.6 (8-14.8)	–	–	–	–
Distance between two phasmids	–	16.8 ± 3.4 (13-24.4)	–	20 ± 3.2 (17-24)	–	24.4 ± 0.3 (15-34)	–	78 ± 16 (58-108)

**Note:** All measurements are in μm (except for ratio) and in the form: mean±s.d. (range).

### Morphological characterization

(Figure 1)

**Figure 1:**
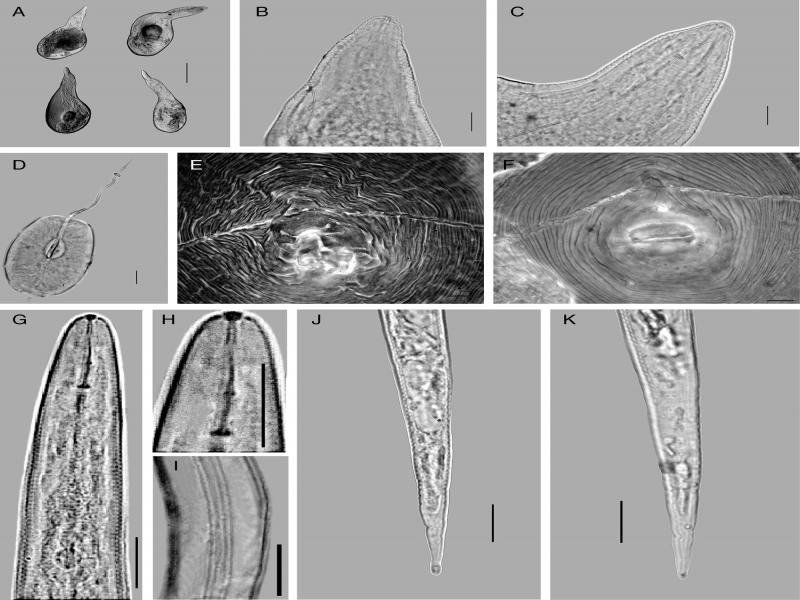
*Meloidogyne javanica* (Chitwood, 1949; Treub, 1885) from *Zingiber officinale* Roscoe and *Eryngium foetidum* L. in Vietnam. A to F: Females. A: Entire body; B, C: Anterior end regions; D: Stylet and median bulb extracted from female; E, F: Perineal patterns. G to K: Second-stage juvenile. G, H: Anterior end region; I: Lateral field; J, K: Tail regions. (Scale bar: A: 200 µm; B-K: 10 µm).

Females: females of *M. javanica* in this study can be characterized by the following features: body pearly white, pear-shaped; lip region offset from body contour with two lip annuli; stylet robust, straight or slightly curved ventrally with rounded or oval stylet knobs directed backwardly; perineal pattern with two prominent lateral lines, dorsal arch squared and slightly narrowed or rounded; striae smooth.

Males: not found.

Second-stage juveniles: second-stage juveniles of *M. javanica* in this study can be characterized by: vermiform body tapering at both ends; slender stylet with rounded and small stylet knobs; secretory-excretory pore located behind level of median bulb; very long pharyngeal gland overlapping intestine ventrally; tail end pointed with rounded tail tip.

### **M**olecular characterization

The Multiplex-PCR amplification products of *M. javanica* in this study were 670 bp, which is in agreement with the study of Kiewnick et al. (2013). The D2-D3 fragment of rDNA sequences of *M. javanica* from this study were 733 bp long and differ only 1 to 2 bp from other sequences of *M. javanica* in GenBank, while the ITS rDNA sequences of *M. javanica* from this study were 477 to 519 bp long and 100% similar to other sequences of *M. javanica* (accession number: JQ917440 and AY829374) from Iran and Spain.

**Figure 2:**
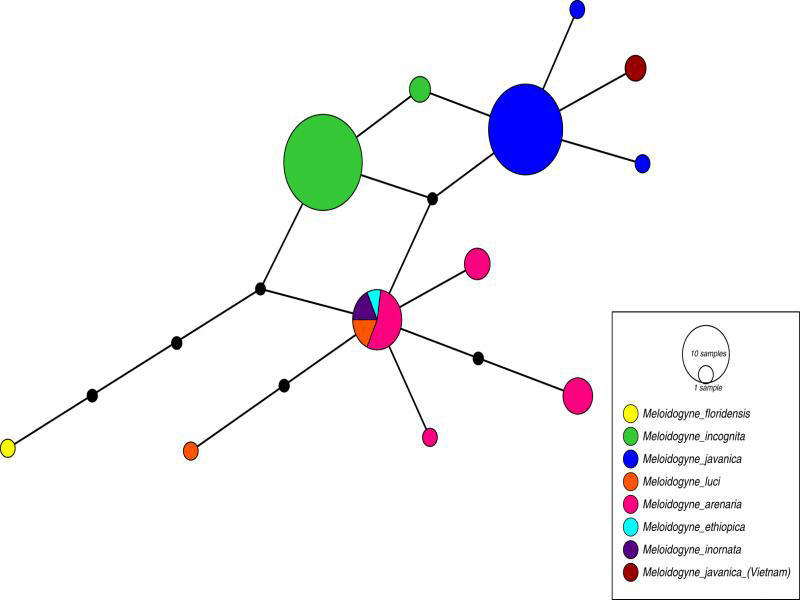
The haplotype network shows the relationships between different haplotypes, circle size is equivalent to the number of studied populations and branch length is equivalent to the number of mutations (shown as small black circles). All populations from the same species are displayed by the same colors (except for the populations of *M. javanica* from Vietnam in this study).

*Nad5* mtDNA sequences of *M. javanica* in this study were obtained with the length from 591 to 609 bp. The sequences of *M. javanica* from ginger and long coriander showed no variation compared to each other. In the network haplotype analysis, sequences of *M. javanica* from ginger and long coriander in Vietnam were grouped together and were only closely with other reference sequences of *M. javanica* from Janssen et al. (2016) (Fig. 2). The identification using PPNID program of Qing et al. (2020) has also confirmed that our studied root-knot nematodes belong to *M. javanica*.

## Remarks and discussion

Morphology and morphometrics of *M. javanica* in this study are largely in agreement with the description of *M. javanica* by Whitehead (1968), except for significant variations in body length and body width of females. However, it is well known that there exist considerable variations in measurements of adult root-knot nematodes between different populations because of their great body size (Whitehead, 1968; Ghaderi et al., 2020). Besides, morphology and morphometric of Vietnamese populations of *M. javanica* are also among variations of *M. javanica* from other countries (Table 1). In general, there exist morphological and genetic variations among populations of *M. javanica* and such variations are rarely inﬂuenced by geographical origin of nematodes and/or their host plants.

In spite of the high similarity between D2-D3 fragment of rDNA and ITS rDNA sequences of *M. javanica* in this study compared to sequences of *M. javanica* from GenBank, it is difficult to distinguish root-knot nematodes in the tropical group using only these DNA regions (Janssen et al., 2016). This study applied a haplotype network analysis based on the *Nad5* sequences to determine the relationship between our nematode populations and the species in the tropical group. The closely related relationship between sequences from our study and reference sequences from Janssen et al. (2016) showed that our nematode populations should belong to *M. javanica*, and this identification was clearly supported by PPNID program of Qing et al. (2020). The results of this study showed the usefulness of the *Nad5* haplotype-based designation as a valuable molecular tool for the identification of tropical root-knot nematode species (Ali et al., 2016; Janssen et al., 2016). Although the integrated approach used in studies of Janssen et al. (2016) and up-to-date authentic barcoding sequences used in PPNID of Qing et al. (2020) are reliable sources for identification of root-knot nematodes, we recommend using multiple approaches in identifying root-knot nematodes to ensure the quality of final result, especially the use of *Nad5* mtDNA gene is recommended for identifying tropical root-knot nematodes.
